# A randomized trial of amifostine in patients with high-dose VIC chemotherapy plus autologous blood stem cell transplanation[Fn tlnote1]

**DOI:** 10.1054/bjoc.2000.1611

**Published:** 2001-02

**Authors:** J T Hartmann, A von Vangerow, L M Fels, S Knop, H Stolte, L Kanz, C Bokemeyer

**Affiliations:** Dept. of Hematology and Oncology, UKT-Medical Center II, Eberhard-Karls-University of Tübingen, Tübingen, 72076; Division of Nephrology, University Medical School Hannover, Hannover, 30625; Society for Science and Technology Transfer (GWT e.V.), Gosen, Berlin, 15537, Germany

**Keywords:** toxicity, high-dose chemotherapy, PBSC transplantation, cytoprotection, amifostine, pharmacoeconomics

## Abstract

This pilot study evaluates the degree of side effects during high-dose chemotherapy (HD-VIC) plus autologous bone marrow transplant (HDCT) and its possible prevention by the cytoprotective thiol-derivate amifostine. Additionally, the in-patient medical costs of both treatment arms were compared. 40 patients with solid tumours were randomized to receive HD-VIC chemotherapy with or without amifostine (910 mg/m^2^ at day 1–3) given as a short infusion prior to carboplatin and ifosfamide. Patients were stratified according to pretreatment. HDCT consisted of an 18 h infusion of carboplatin (500 mg/m^2/^d over 18 h), ifosfamide (4 g/m^2^/d over 4 h) and etoposide (500 mg/m^2^/d) all given for 3 consecutive days. All patients received prophylactic application of G-CSF (5 μg kg^−1^ subcutaneously) to ameliorate neutropenia after treatment. Patients were monitored for nephrotoxicity, gastrointestinal side effects, haematopoietic recovery, as well as frequency of fever and infections. The median fall of the glomerular filtration rate (GFR) was 10% from baseline in the amifostine group (105 to 95 ml min^−1^) and 37% in the control patient group (107 to 67 ml min^−1^) (*P*< 0.01). Amifostine-treated patients revealed a less pronounced increase in albumine and low molecular weight protein urinary excretion. Stomatitis grade III/IV occurred in 25% without versus 0% of patients with amifostine (*P* = 0.01). Acute nausea/vomiting was frequently observed immediately during or after the application of amifostine despite intensive antiemetic prophylaxis consisting of 5-HT_3_-receptor antagonists/dexamethasone/trifluorpromazine. However, delayed emesis occurred more often in the control patients. Engraftment of neutrophil (> 500 μl^−1^) and thrombocytes (> 25 000 μl^−1^)were observed at days 9 versus 10 and 10 versus 12, respectively, both slightly in favour of the amifostine arm. In addition, a lower number of days with fever and a shortened duration of hospital stay were observed in the amifostine arm. The reduction of acute toxicity observed in the amifostine arm resulted in 30% savings in costs for supportive care (Euro 4396 versus Euro 3153 per patient). Taking into account the drug costs of amifostine, calculation of in-patient treatment costs from the start of chemotherapy to discharge revealed additional costs of Euro 540 per patient in the amifostine arm. This randomized pilot study indicates that both organ and haematotoxicity of HD-VIC chemotherapy can be ameliorated by the use of amifostine. Additionally, a nearly complete preservation of GFR was observed in amifostine-treated patients which may be advantageous if repetitive cycles of HDCT are planned. Larger randomized trials evaluating amifostine cytoprotection during high-dose chemotherapy are warranted. © 2001 Cancer Research Campaign http://www.bjcancer.com


*British Journal of Cancer* (2001) **84**(3), 313–320
© 2001 Cancer Research Campaign
doi: 10.1054/ bjoc.2000.1611, available online at http://www.idealibrary.com on IDEAL®

http://www.bjcancer.com

### A randomized trial of amifostine in patients with high-dose VIC chemotherapy plus autologous blood stem cell transplanation*

**JT Hartmann[1], A von Vangerow[1], LM Fels[2,3], S Knop[1], H Stolte[2,3], L Kanz[1] and C Bokemeyer[1]**

[1]Dept. of Hematology and Oncology, UKT-Medical Center II, Eberhard-Karls-University of Tübingen, 72076 Tübingen, [2]Division of Nephrology, University Medical School Hannover, 30625 Hannover, [3]Society for Science and Technology Transfer (GWT e.V.), Gosen, 15537 Berlin, Germany

**Summary** This pilot study evaluates the degree of side effects during high-dose chemotherapy (HD-VIC) plus autologous bone marrow transplant (HDCT) and its possible prevention by the cytoprotective thiol-derivate amifostine. Additionally, the in-patient medical costs of both treatment arms were compared. 40 patients with solid tumours were randomized to receive HD-VIC chemotherapy with or without amifostine (910 mg/m$^2$ at day 1–3) given as a short infusion prior to carboplatin and ifosfamide. Patients were stratified according to pretreatment. HDCT consisted of an 18 h infusion of carboplatin (500 mg/m$^{2/}$d over 18 h), ifosfamide (4 g/m$^2$/d over 4 h) and etoposide (500 mg/m$^2$/d) all given for 3 consecutive days. All patients received prophylactic application of G-CSF (5 µg kg$^{-1}$ subcutaneously) to ameliorate neutropenia after treatment. Patients were monitored for nephrotoxicity, gastrointestinal side effects, haematopoietic recovery, as well as frequency of fever and infections. The median fall of the glomerular filtration rate (GFR) was 10% from baseline in the amifostine group (105 to 95 ml min$^{-1}$) and 37% in the control patient group (107 to 67 ml min$^{-1}$) ($P < 0.01$). Amifostine-treated patients revealed a less pronounced increase in albumine and low molecular weight protein urinary excretion. Stomatitis grade III/IV occurred in 25% without versus 0% of patients with amifostine ($P =$ 0.01). Acute nausea/vomiting was frequently observed immediately during or after the application of amifostine despite intensive antiemetic prophylaxis consisting of 5-HT$_3$-receptor antagonists/dexamethasone/trifluorpromazine. However, delayed emesis occurred more often in the control patients. Engraftment of neutrophil (> 500 µl$^{-1)}$ and thrombocytes (> 25 000 µl$^{-1)}$ were observed at days 9 versus 10 and 10 versus 12, respectively, both slightly in favour of the amifostine arm. In addition, a lower number of days with fever and a shortened duration of hospital stay were observed in the amifostine arm. The reduction of acute toxicity observed in the amifostine arm resulted in 30% savings in costs for supportive care (Euro 4396 versus Euro 3153 per patient). Taking into account the drug costs of amifostine, calculation of in-patient treatment costs from the start of chemotherapy to discharge revealed additional costs of Euro 540 per patient in the amifostine arm. This randomized pilot study indicates that both organ and haematotoxicity of HD-VIC chemotherapy can be ameliorated by the use of amifostine. Additionally, a nearly complete preservation of GFR was observed in amifostine-treated patients which may be advantageous if repetitive cycles of HDCT are planned. Larger randomized trials evaluating amifostine cytoprotection during high-dose chemotherapy are warranted. © 2001 Cancer Research Campaign http://www.bjcancer.com

**Keywords**: toxicity; high-dose chemotherapy; PBSC transplantation; cytoprotection; amifostine; pharmacoeconomics

High-dose chemotherapy with autologous haematopoetic stem cell transplantation (HDCT) has been used in selected patients with chemosensitive solid tumours and haematologic neoplasms. Randomized trials have indicated an improved outcome with HDCT in comparison to standard treatment in patients with relapsed high-grade non-Hodgkin's lymphoma, advanced multiple myeloma, and acute myelogenous leukaemia after remission induction without allogenic bone marrow donor (Philip et al, 1995; Zittoun et al, 1995; Attal et al, 1996). The role of HDCT is currently investigated in poor-risk high-grade non-Hodgkin's lymphoma (Gianni et al, 1997a; Haioun et al, 1997), relapsed Hodgkin's disease (Linch et al, 1993), breast (Gianni et al, 1997b; Rizzieri et al, 1999) and ovarian cancer, SCLC as well as 'poor prognosis' or relapsed germ cell tumours (Siegert et al, 1994; Savarese et al, 1997; Hartmann et al, 1999a). The combination of high-dose carboplatin, etoposide and ifosfamide has been developed as an active regimen for a variety of malignancies (Wright et al, 1995; Beyer et al, 1997).

Amifostine, WR-2721, an organic thiophosphate cytoprotective agent, is a prodrug which is dephosphorylated to its active metabolite, WR-1065, by tissue-bound alkaline phosphatase. WR-1065 acts via different mechanisms including radical scavening, hydrogene donation and in the case of platinum compounds, prevention or reversal of platinum-DNA adducts. HDCT regimen containing carboplatin, etoposide and ifosfamide is associated with multiple side effects such as haematologic and non-haematologic toxicities, e.g. acute nephrotoxicity and mucosal damage (Siegert et al, 1994; Elias et al, 1995; Wright et al, 1995; Beyer et al, 1997). The rationale for the use of amifostine in a controlled clinical trial in patients receiving HDCT is based on the exclusion of a significant pharmacokinetic interaction between amifostine and cytostatics

*Received 21 June 2000*
*Revised 5 October 2000*
*Accepted 27 October 2000*

*Correspondence to*: C Bokemeyer

* Presented in part at the 35th Proceedings of the American Society of Clinical Oncology, Atlanta, Georgia, USA, May 15–18, 1999. This work represents part of the thesis of A. von Vangerow at the Dept. Hematology/Oncology, University of Tübingen.

(Treskes and van der Vijgh, 1993; Gelmon et al, 1999), and the evidence of its broad spectrum cytoprotective properties resulting in a reduction of both haematologic and organ toxicities without tumour protection (Kemp et al, 1996; Elias et al, 2000; Hartmann et al, 2000a, 2000b). Preservation of renal function using the combination of cisplatin, ifosfamide and etoposide plus amifostine had been previously demonstrated (Hartmann et al, 1999b). Based on this background a randomized pilot study was conducted to evaluate the degree of side effects during HDCT and its possible prevention by amifostine in patients with solid tumours. In-patient costs for both treatment arms were compared in detail.

### MATERIAL AND METHODS

### Inclusion and exclusion criteria

Criteria for inclusion were required as follows: ECOG performance status of 0 or 1, adequate bone marrow function (WBC-count $\geqslant$ 4000 $\mu l^{-1}$, platelet-count $\geq$ 100 000 $\mu l^{-1}$ and granulocyte count $\geq$ 2000 $\mu l^{-1}$), adequate renal (serum creatinine concentration $\leq$ 1.2 mg $dl^{-1}$ and creatinine clearance $\geq$ 80 ml $min^{-1}$) and liver function (bilirubin level $\leq$ 2.0 mg $dl^{-1}$, and AST and ALT $\leq$3 times normal). Parameters for ineligibility included presence of brain metastases, symptoms of ischaemic heart disease, and history of congestive heart failure or myocardial infarction within the immediate preceding 6 months and/or clinically significant arrhythmia.

Patients had given informed consent both for the treatment with HD-VIC and for the randomization to pretreatment with or without amifostine. Patients were stratified according to the absence or presence of prior chemotherapy. This study had been approved by the local University ethical committee.

### Treatment schedule

All patients were randomized to receive HD-VIC chemotherapy plus PBSC transplant and granulocyte colony-stimulating factor (G-CSF) support either with or without amifostine pretreatment during HDCT as outlined in Table 1. HDCT consisted of an simultaneous 18 h infusion of carboplatin 500 mg/m$^2$/d and ifosfamide 4 g/m$^2$/d preceded by a 4 h infusion of etoposide 500 mg/m$^2$/d, all applied on three consecutive days (Fetscher et al, 1999).

**Table 1** HD-VIC chemotherapy schedule

| | |
|---|---|
| Agents | V: Etoposide 500 mg/m$^2$ (4 h) per day (d1–3)[b] |
| Dose | I: Ifosfamide 4000 mg/m$^2$ (18 h) per day (d1–3)[c] |
| Infusion schedule | C: Carboplatin 500 mg/m$^2$ (18 h) per day (d1–3)[c] |
| Mesna[a] | 4 g/m$^2$/24 h[d] |
| (mercaptoethanesulfonate) | day 1–3 |
| Haematopoetic support | G-CSF from d 5 (5 µg/kg s.c) until neutrophil count >5000 $\mu l^{-1}$ |
| Hydration | 2000 ml isotonic NaCl/m$^2$ per 24 h, day 1–3<br>60 mmol magnesium and 6.9 mmol calcium per 24 h, day 1–3 |
| PBSC transplantation | at day 5 (minimum of 2 $\times$ 10$^6$ CD34+ cells/kg body weight) |
| Amifostine dose (arm A) | |
| Infusion time | 910 mg/m$^2$/per day (d1–3) 15 min-infusion (prior to carboplatin/ifosfamide) |

G-CSF = Granulocyte-colony stimulating factor. [a]To prevent ifosfamide-induced hemorrhagic cystitis. [b]Solved in 2000 ml NaCl 0.9%. [c]Solved in 500 ml NaCl 0.9%. [d]Continued for 12 hours after the end of infusion.

Antiemetic prophylaxis consisted of 5-HT$_3$-antagonists and dexamethasone (20 mg i.v.) administrated 30 minutes prior to chemotherapy application. Dexamethasone 4 mg was repeated at 4 and 12 hours after the start of chemotherapy. Patients treated with amifostine additionally received 10 mg trifluorpromazine intravenously at each day of treatment. All patients received autologous PBSC transplantation two days after HDCT and G-CSF at a dose of 5 µg $kg^{-1}$ body weight as a subcutaneously injection starting at day +5 until the granulocyte count was >5000 $\mu l^{-1}$ (Table 1). Patients were monitored for nephrotoxicity, gastrointestinal side effects, haematopoetic recovery, frequency of fever/infections, and days spent in hospital. All patients underwent daily blood counts, serum electrolytes and creatinine measurements and a careful clinical examination. Creatinine clearance was measured immediately before and at day 10 after the application of HDCT. Patients were stratified according to pretreatment (none versus $\geq$ 1 chemotherapeutic regimen/s) and the number of reinfused PBSC (<2.9 or $\geq$ 3.0 $\times$ 10$^6$ CD34+ cells/kg body weight).

### Supportive treatment

All patients received ofloxacin 200 mg orally twice daily and fluconazol 100 mg orally daily as antibacterial and antifungal prophylaxis starting at the day of treatment and continued unless intravenous antibiotic treatment was required in case of fever. Empiric combination treatment of a double-lactam penicilline and an aminoglycoside antibiotic was started when temperatures exceed 38.5˚C. The antibiotic regimen was modified according to microbiologic findings and clinical requirements. Patients were not nursed in reverse-barrier isolation. Total parenteral nutrition was given during periods of inadequate oral calorie intake. Following HD-VIC it was routine practice to transfuse red blood cell concentrates to maintain haemoglobin-levels higher than 8 g $dl^{-1}$ and platelet concentrates to maintain platelet count $\geq$ 15 000 $l^{-1}$.

### Application of amifostine

Amifostine was applied at 910 mg/m$^2$ as a 15-min intravenous infusion completed 15 min before the start of the simultaneous carboplatin and ifosfamide infusion at each of the three consecutive days of treatment. Blood pressure was monitored every 5 min during the amifostine infusion. Amifostine was interrupted in case of a >20 mmHg decrease in systolic blood pressure depending on the patients baseline blood pressure level. Antihypertensive medication was withheld within 24 hours after amifostine administration.

### Urine sample collection and measurement of urinary marker excretion

Urine samples were collected immediately prior to HDCT (day−1) and on days 3, 5 and 10 after HDCT. Parameters of glomerular and tubular renal function such as total protein, albumine fraction, low molecular weight proteins (LMW), high molecular weight proteins (HMW), $\alpha_1$-microglobulin and N-acetyl-$\beta^2$ glucosaminidase (NAG) were determined from the urinary samples as described earlier (Hartmann et al, 2000b). Ratios for creatinine clearance ($C_{creatinine}$) were calculated using the general clearance formula for a substance y: $clearance_y = ([urine]_y/[plasma]_y) \times$ urine volume.

 © 2001 Cancer Research Campaign

### Measurement of toxicity

Except for nephrotoxicity which was also characterized by the measurement of creatinine clearance and the degree of urinary protein excretion, side effects of treatment were classified according to WHO toxicity scale. In order to minimize observation bias the degree of toxicity of the gastrointestinal tract, such as nausea, vomiting, stomatitis and diarrhoea, was judged by the same person during the study period. For statistical calculation the highest toxicity degree occurring during HDCT was used to compare the both study arms.

### Calculation of treatment costs

Detailed financial accounts of charges for each patient included into the study were reviewed from the beginning of chemotherapy until discharge. Charges were converted from German marks (DEM) to Euro (Euro 1 = DEM 1.95). The cost analysis was based on average wholesale price for each resource in Germany. Prices for alimentation and transfusions were obtained from the billing records of the University Medical Center Tübingen. The economic calculation was performed on the basis of direct medical costs (including drugs, costs of hospitalization, diagnostic radiology, labaratory tests, cultures, pharmacy, blood products) from a third party payer perspective from the start of treatment to discharge (Eisenberg, 1989; Russel et al, 1996). However, the costs for nursing and professional time as well as monitoring devices associated with care for severely ill patients were not considered separately because these costs are included in the costs of hospitalization in the German health system. Indirect costs, f.e. decreased work productivity, or costs savings due to a different degree of late side effects were also not considered in the analysis. Patients were discharged in case of independence from platelet transfusions defined as a thrombocyte count >25 000 $\mu l^{-1}$ on two consecutive days and recovery of neutrophil >1500 $\mu l^{-1}$, absence of fever and complete recovery from other grade III/IV toxicity.

### Statistics

Non-parametric parameters of haematologic, renal, gastrointestinal toxicity as well as changes in the excretion rate of urinary analytes were compared using the Mann-Whitney U-Test. Statistical analysis was performed with SPSS 6.0 (SPSS, Chicago, IL, USA). Significance was defined as $P \leq 0.05$. All reported *P* values were two-sided. Friedman-Oneway-ANOVA procedures were applied in the follow-up of arms to evaluate whether the variance of the excretion rates of the urinary analytes was influenced by the time point of collection.

### RESULTS

### Patients' characteristics

40 consecutive patients receiving HD-VIC plus autologous PBSC transplantation for different solid tumours between August 1997 and January 1999 were enrolled into the study and were followed from start of treatment until discharge. Patients' characteristics are listed in Table 2. Patients in both arms were comparable in terms of pretreatment due to stratification. Of 40 patients randomized either to the amifostine arm or to the control arm, 26 (65%) had received no previous chemotherapy except for two cycles of induction chemotherapy (VIP-regimen consisting of etoposide, ifosfamide, cisplatin) to collect PBSC before the application of the HD-VIC therapy examined here (Fetscher et al, 1999). While 6 patients in the amifostine arm had previously treated with chemotherapy regimens containing cisplatin or ifosfamide, none of the patients belonging to the control arm had received these types of chemotherapy. The majority of patients had metastatic breast cancer and had been treated with HDCT according to the GEPDIS protocol (German Breast Cancer Dose Intensity Study).

**Table 2** Patients' characteristics

| | Amifostine | Control arm |
|---|---|---|
| N (pts) randomized | 20 | 20 |
| Sex | | |
| male/female | 4/15 | 0/20 |
| Median age | | |
| years (range) | 42 (30–58) | 48 (36–62) |
| Type of solid tumours | breast cancer (*n* = 14) | breast cancer |
| (N pts) | testicular cancer (*n* = 3) | (*n* = 20) |
| | sarcoma (*n* = 2) | |
| | ovarian cancer (*n* = 1) | |
| Previous chemotherapy (N pts)[a] | 7 (35%) | 7 (35%) |
| cisplatin-containing (N pts) | 4 (20%) | – |
| mean cisplatin dose (range) | 400 mg/m² (300–500) | – |
| ifosfamide-containing (N pts) | 2 (10%) | – |
| mean ifosfamide dose (range) | 9 g/m² (6–12) | – |

[a]In addition to standard VIP induction chemotherapy for PBSC mobilization.

### Nephrotoxicity

All 40 patients were evaluable for serum creatinine, creatinine clearance (GFR = glomerular filtration rate) and magnesium values, and for urinary marker excretion analysis. Both arms had received a comparable volume load of either saline or saline plus glucose solution, as well as a comparable use of diuretics. No differences existed in baseline parameters (day -1) between both arms ($P > 0.05$) except for the magnesium serum and creatinine serum concentrations both in favour of the control group ($P = 0.02$ and $P = 0.02$). Patients treated with HD-VIC plus amifostine revealed a significantly lower degree of renal damage compared to the control arm. Following HDCT the median GFR values decreased by 37% from 107 ml $min^{-1}$ (63–196) at baseline to 67 ml $min^{-1}$ (30–148) at day 10 in control patients. In contrast, in the amifostine arm the GFR was almost completely preserved with 102 ml $min^{-1}$ (60–149) prior to and 92 ml $min^{-1}$ (51–133) after application of chemotherapy ($P < 0.01$). 60% of patients treated with HD-VIC alone had a GFR < 60 ml $min^{-1}$ at day 10 after treatment compared to 20% of patients in the amifostine arm (Table 3).

The median decrease of serum magnesium levels was 8% in patients with HD-VIC plus amifostine versus 17% in patients without amifostine. However, the nadirs of Mg values were comparable due to different serum concentrations prior to the start of chemotherapy between both treatment arms. Magnesium levels have completely recovered in both groups at day 10 after treatment.

The determination of urinary markers revealed significant nephrotoxic effects in both treatment arms. Protein excretion for all fractions was increased about 20 to 35-fold by days 3 and 5 in both arms. There were no signs of any pre- or post-renal causes for these changes, such as e.g. haemorrhagic cystitis. An electrophoretic separation of the proteins excreted showed that the

**Table 3** Parameters of renal toxicity in solid tumour patients following HD-VIC chemotherapy ± amifostine

| | **Amifostine ($n$ = 20)** | **Control ($n$ = 20)** | ***P* value[a]** |
|---|---|---|---|
| Serum creatinine (mg dl$^{-1}$) (range) | | | |
| Baseline | 0.8 (0.6–1.3) | 0.7 (0.5–0.9) | 0.02 |
| Peak | 0.9 (0.7–1.5) | 1.1 (0.7–1.6) | 0.5 |
| Median increase (%) | 12 (0–87) | 41 (14–100) | 0.03 |
| GFR (ml/min) [range] | | | |
| Baseline | 102 (60–140) | 107 (63–196) | 0.5 |
| Day 10 | 92 (51–133) | 67 (30–148) | <0.01 |
| Fall of GFR from baseline (%) | 10 (0–47) | 37 (17–69) | <0.01 |
| % of pts with GFR < 80 ml min$^{-1}$[b] | 20 | 60 | <0.01 |
| % of pts with GFR < 60 ml min$^{-1}$[b] | – | 35 | <0.01 |
| Serum magnesium (mmol$^{-1}$ l) (range) | | | |
| Baseline | 0.75 (0.67–0.87) | 0.82 (0.73–0.94) | 0.02 |
| Nadir | 0.69 (0.65–0.80) | 0.66 (0.49–1.20) | 0.1 |
| Fall of serum magnesium (%) | 8 (0–27) | 17 (6–43) | 0.01 |

All values given in median and standard deviation. [a] = Mann-Whitney U-test. pts = patients.
GFR = creatinine clearance in ml/min (baseline to day 10). [b] After HDCT.

**Table 4** Excretion of urinary marker excretion following of HD-VIC regimen ± amifostine

| | **Amifostine (median, range)** | **Control (median, range)** | ***P* value[a]** |
|---|---|---|---|
| a1-microglobulin (mg g$^{-1}$ creatinine) | | | |
| Day 1 | 5 (0–63) | 23 (1–45) | n.s. |
| 3 | 71 (29–182) | 124 (26–345) | n.s. |
| 5 | 92 (37–197) | 80 (29–335) | n.s. |
| P value[b] | <0.001 | <0.001 | |
| NAG (U g$^{-1}$ creatinine) | | | |
| Day 1 | 2 (1–5) | 3 (1–16) | n.s. |
| 3 | 10 (3–31) | 16 (0–60) | n.s. |
| 5 | 17 (6–89) | 14 (8–50) | n.s. |
| P value[b] | 0.001 | 0.001 | |
| Total protein (mg g$^{-1}$ creatinine) | | | |
| Day 1 | 59 (31–247) | 108 (34–575) | n.s. |
| 3 | 296 (131–566) | 399 (85–838) | n.s. |
| 5 | 380 (18–2617) | 275 (132–949) | n.s. |
| P value[b] | 0.001 | 0.001 | |
| LMW (mg g$^{-1}$ creatinine) | | | |
| Day 1 | 23 (4–203) | 48(2–132) | n.s. |
| 3 | 206 (69–405) | 243 (48–541) | n.s. |
| 5 | 208 (26–860) | 130 (64–569) | n.s. |
| P value[b] | 0.001 | 0.001 | |
| HMW (mg g$^{-1}$1 creatinine) | | | |
| Day 1 | 18 (31–247) | 16 (5–281) | n.s. |
| 3 | 31 (7–57) | 57 (13–123) | 0.009 |
| 5 | 52 (5–171) | 61 (16–112) | n.s. |
| P value[b] | n.s. | 0.001 | |
| Albumine (mg g$^{-1}$ creatinine) | | | |
| Day 1 | 13 (1–35) | 11 (3–73) | n.s. |
| 3 | 30 (11–81) | 50 (13–87) | n.s. |
| 5 | 45 (5–398) | 48 (12–286) | n.s. |
| P value[b] | 0.001 | 0.002 | |

NAG = N-acetyl-β-glucosaminidase; LMW = low molecular weight proteins; HMW = high molecular weight proteins. [a]=Mann-Whitney U-test. [b]=ANOVA procedure.

excretion of all three protein fractions, HMW proteins, albumin and LMW proteins, was significantly elevated following HDCT, as well as $\alpha_1$-microglobulin and NAG as markers of a proximal tubular damage. The excretion of LMW proteins at $day_3$ and albumine at $days_{3,5,10}$ was significantly lower in the amifostine arm compared to control patients ($P$ < 0.05) (Table 4).

### Haematotoxicity

All patients experienced WHO grade IV haematotoxicity followed by a full haematologic reconstitution. Granulocyte counts > 500 µl after PBSC-transplantation were observed at day 9 (range, 7–12) in the amifostine arm and at day 10 (range, 8–13) in the control

 *© 2001 Cancer Research Campaign*

**Table 5** Parameters of haematotoxicity and recovery of blood cell counts in patients with HD-VIC chemotherapy ± amifostine

| | Amifostine (*n* = 20) | Control (*n* = 20) | *P* value[a] |
|---|---|---|---|
| Neutrophil engraftment > 500 µl$^{-1}$ Median N of days (range) | 9 (7–12) | 10 (8–13) | 0.02 |
| Thrombocyte engraftment >25 000 µl$^{-1}$ Median N of days (range) | 10 (9–17) | 12 (10–17) | 0.01 |
| RBC transfusions Median Units (range) | 2 (0–4) | 2 (0–6) | 0.1 |
| Platelet transfusions Median Units (range) | 3 (1–5) | 3 (1–7) | 0.08 |
| No. of reinfused PBSC (x10$^6$ CD34+ cells/kg body weight) | 2.8 (2.0–3.9) | 3.0 (2.2–7.8) | 0.3 |
| Occurrence of fever in neutropenia (> 38.5˚C) Median N of days (range) | 2 (0–5) | 4 (0–7) | 0.03 |
| N of pts requiring antibiotis [%][b] | | | |
| None | 3 (15) | 2 (10) | |
| First-line regimen | 15 (75) | 7 (35) | |
| Salvage regimen | 2 (10) | 11 (55) | 0.03 |

[a] = Mann-Whitney U-test. [b] = in case of fever >38.5˚C. RBC = red blood cell; PBSC = peripheral blood stem cells.

arm (*P* = 0.02). Both arms have received additional treatment with G-CSF s.c. Thrombocyte engraftment (> 25 000 µl$^{-1}$) was reached at a median of two days earlier in the amifostine arm (*P* = 0.01). However, patients in both groups needed platelet transfusions after HD-VIC. The number of reinfused PBSC was comparable for both arms (2.8 (range, 2.0–3.9) versus 3.0 × 10$^6$ CD34+ cells µl$^{-1}$ (range, 2.2–7.8); *P* = 0.3). The median number of days with fever > 38.5˚C was 2 (range, 0–5) in amifostine pretreated patients versus 4 days (range, 0–7) in control patients (*P* = 0.03) (Table 5).

### Gastrointestinal toxicity

All patients had routinely received 5-HT$_3$-receptor antagonists and dexamethasone as antiemetic prophylaxis. Patients treated with amifostine additionally received 10 mg trifluorpromazine intravenously at each day of treatment (days 1–3). Some degree of acute nausea shortly after or during the application of amifostine was observed in all patients (see side effects of amifostine). However, the proportion of patients with delayed nausea/vomiting (defined as beyond 24 hours after completion of chemotherapy) grade ≤III was higher in the control arm resulting in a overall lower rate of antiemetics applied. Both stomatitis and diarrhoea WHO grade III/IV was observed in 25% of patients with amifostine and in half of the patients without amifostine (Table 6).

Side effects associated with the use of amifostine were acute short-lasting nausea/vomiting observed after or during the administration of amifostine, a mild self-limited rash (20/20 cycles = 100%), sneezing (30%), hypotension (= blood pressure decrease > 20 mmHg) without requiring medical intervention (25%), hiccups (15%) and mydriasis (5%). Mild and asymptomatic hypocalcaemia was observed in 80% of patients (*n* = 12/15 evaluable) in the amifostine and 73% of patients in the control arm (*n* = 11/15 evaluable). The mean decrease of serum magnesium was 16% (range, 9–26%) in the amifostine arm at day 4 (range, 3–10) and 13% (range, 4–24%) in the control patients at day 7 (range, 3–13). All patients had routinely received intravenous calcium supplementation during treatment (see Table 1). There was no treatment-related mortality observed in both arms.

**Table 6** Gastrointestinal toxicity in patients with HD-VIC chemotherapy ± amifostine

| | Amifostine (*n* = 20) | Control (*n* = 20) | *P* value[a] |
|---|---|---|---|
| Delayed nausea/vomiting (>24 hours) | | | |
| Median WHO grade (range) | 2 (1–3) | 3 (2–4) | |
| III/IV (% of pts.) | 25 | 85 | <0.01 |
| Stomatitis | | | |
| Median WHO grade (range) | 2 (1–3) | 3 (1–4) | |
| III/IV (% of pts.) | 25 | 50 | 0.01 |
| Diarrhoea | | | |
| Median WHO grade (range) | 1 (0–3) | 2.5 (0–3) | |
| III/IV (% of pts) | 25 | 50 | 0.05 |

[a] = Mann-Whitney U-test.

### In-patient cost calculation

Pretreatment with amifostine resulted in a reduced use of antibiotics, diagnostic radiology and cultures, platelet and red blood cell transfusions, as well as of parenteral nutrition compared to control patients. Despite a higher rate of acute nausea and emesis observed immediately during or after the infusion of amifostine, an increased frequency of delayed nausea and vomiting resulted in higher costs for antiemetic medication in the control arm as stated before. Patients treated with amifostine were discharged from hospital at day 16 compared to day 18 in the control arm (*P* < 0.01). Therefore, costs for laboratory and hospitalization were lower.

Overall costs for supportive care were Euro 63 063 (Euro 3153 per patient) in the amifostine arm versus Euro 87 931 (Euro 4396

**Table 7** Cost of treatment for supportive care in patients with HD-VIC chemotherapy ± amifostine – (in Euro)

| Costs for supportive care products | Amifostine (*n* = 20) | Control arm (*n* = 20) |
|---|---|---|
| Antiemetics | 1966 | 2932 |
| Antibiotics | 8844 | 11 088 |
| G-CSF | 19 209 | 19 779 |
| Platelet transfusions | 17 116 | 21 913 |
| Red blood cell packages | 2382 | 2693 |
| Diagnostic radiology | 531 | 715 |
| Cultures | 5091 | 6980 |
| Labaratory | 7754 | 8861 |
| Parenteral nutrition | 170 | 293 |
| Incremental hospitalization | – | 12 675 |
| Total | 63 063 | 87 931 |
| Total per patient | 3153 | 4396 |

© 2001 Cancer Research Campaign per patient) in the control arm (not considering the additional costs of the drug amifostine). Thus, savings of supportive care costs in the amifostine arm were Euro 1243 per patient. Taking into account the drug costs of amifostine (Euro 1783 per patient), treatment with amifostine resulted in additional costs of Euro 540 per patient compared to the control arm (Table 7).

### DISCUSSION

High-dose chemotherapy (HDCT) with autologous peripheral blood stem cell transplantation has been used for defined subgroups of patients with chemosensitive malignancies. The escalation of drugs such as etoposide and ifosfamide and the use of high-dose carboplatin has become possible through the management of haematotoxicity with the availability of recombinant haematopoietic growths factors and PBSC techniques. However, with increased dosages of cytostatics non-haematologic organ toxicities have become dose-limiting. Patients treated with regimens like HD-ICE/-VIC/-CEI containing carboplatin, ifosfamide and etoposide are at risk for pronounced gastrointestinal toxicity, infections, acute CNS toxicity and cognitive impairment (Siegert et al, 1994; Elias et al, 1995; Wright et al, 1995; Beyer et al, 1997; van Dam et al, 1998; Fetscher et al, 1999). Additionally, high dose carboplatin, which is not nephrotoxic when applied in standard doses and high-dose ifosfamide can produce significant renal damage (Hartmann et al, 2000c; Wagstaff et al, 1989; van Dam et al, 1998). The level of side effects may be pronounced particularly in patients who have undergone intensive pretreatment. Thus, it is important to evaluate methods to reduce these toxicities for regimens commonly used in the HDCT setting.

In clinical practice nephrotoxicity is measured via creatinine serum values and the determination of the glomerular filtration rate (GFR). The GFR measured by creatinine clearance is a more sensitive parameter to detect glomerular damage compared to serum creatinine values. In this study the GFR was clearly affected in patients receiving HD-VIC without prior application of amifostine. Patients in the control arm had a median loss of GFR of 37% compared to their baseline level after the application of the HDCT cycle, and 35% of these patients had GFR values below 60 ml $min^{-1}$ at day 10 after HDCT. This reduction of GFR may result in clinically relevant long-term effects particularly when further therapy is required in the case of relapse, or when a second cycle of HDCT is planned, or even when other nephrotoxic agents such as aminoglycoside antibiotics have to be used. Among patients receiving amifostine during HD-VIC chemotherapy the GFR value dropped only by a median of 10% and no patient developed GFR-values below 60 ml $min^{-1}$ at day 10. Nephroprotection was also demonstrated by a lower degree of urinary protein excretion–particularly LMW proteins and albumine–compared to patients in the control group. Magnesium and calcium supplementation were used during the hydration period in both treatment arms, and therefore hypomagnesaemia or symptomatic hypocalcaemia were not a clinically evident problem in both groups (Wadler et al, 1993). Although hypocalcaemia was observed earlier in the time course after the application of treatment in amifostine-pretreated patients (day 4 versus 7), the degree and frequency were comparable among both arms.

The degree of nephrotoxicity in the HDCT setting with regimen based on ifosfamide and high-dose carboplatin was not unexpected (Rosti et al, 1992; Barnett et al, 1993; Siegert et al, 1994; Fields et al, 1995; Margolin et al, 1996; Fetscher et al, 1999). Broun and coworkers (1991) have observed renal failure in 4 of 7 patients and gross haematuria in 2 patients. Siegert et al (1994) reported a somewhat lower level of nephrotoxicity. This discrepancy may be explained by the different duration of ifosfamide application. In the first trial (Broun et al, 1991) the drug was applied as a short infusion over 30 minutes, whereas in the study of Siegert et al (1994) it was administrated as continuous infusion over 22 hours, thus avoiding high peak drug concentrations.

In an analysis of 150 consecutively treated patients with relapsed germ cell tumours treated with HD-CEI (carboplatin 1500–2000 mg/$m^2$/etoposide, 1200–2400 mg/$m^2$/ifosfamide 0–10 g/$m^2$) followed by either bone marrow or PBSC rescue, mortality was 3% ($n$ = 5 patients), with 3 patients dying from severe renal toxicity (Beyer et al, 1997). Overall, acute nephrotoxicity occurred in 29% of patients, particularly at carboplatin >1500 mg/$m^2$. Nephrotoxicity was defined as either a decline in the estimated creatinine clearance of ≥50% or as an absolute increase of serum creatinine ≥1 mg $dl^{-1}$. Intermittent haemodialysis was required in 8% of patients, and two patients entered chronic haemodialysis. Nephrotoxicity resulted in an increased needs for transfusions, more overall toxicity and a prolonged hospital stay. Whereas the intensity of previous exposure to cisplatin has been reported as a risk factor for nephrotoxicity from ifosfamide administration in children (Goren et al, 1987), Beyer and coworkers were unable to identify any variables that might predict acute nephrotoxicity after HD-CEI treatment. Two other reports that studied HD-CEI in heterogeneous patient populations described the frequency of acute nephrotoxicity as 46% and 29%, respectively (Wilson et al, 1992; Elias et al, 1995). Empiric dose reduction of HD-CEI has been suggested as soon as the deterioration of renal function becomes apparent in order to prevent further renal damage. The strategy of empiring dose reductions is important and may save patients from irreversible side effects or even death, but, it did not prevent the occurrence of acute nephrotoxicity in about one third of patients. As an alternative method the a priori adjustment of the carboplatin dose according to the patients' renal function has been used to achieve a predefined target area under the curve for this drug (Calvert et al, 1989). However, the formula that was devised by Calvert et al has only been established for single-dosing and single-agent carboplatin administration. It does not take into account the day to day changes that occur with combination chemotherapy including further potentially nephrotoxic drugs and with repetitive dosing. In addition, no GFR-based formula for dose calculations of etoposide and ifosfamide exists.

Renal insufficiency was also the dose-limiting toxicity of the escalation of the HD-ICE regimen (Wright et al, 1995). Wright and coworkers proposed a model of plasma drug level measurement early during treatment to provide warning of renal failure. 9 patients received a 96 h infusion of ifosfamide (16 g/$m^2$), carboplatin (1.6 g/$m^2$) and etoposide (1.2 g/$m^2$). The drug levels and plasma concentration–time curves of ifosfamide, ultrafiltrable platinum and etoposide were analysed and correlated with renal function. One of the 9 patients developed anuric renal failure requiring haemodialysis. The authors conclude that high plasma ifosfamide and ultrafiltrable platinum levels analysed early in the course of the 96 h infusion of HD-ICE may provide a warning of severe and potentially fatal renal injury.

Stomatitis with oesophagitis was common with HD-VIC (Elias et al, 1995). Fields et al (1995) reported a high incidence of enteritis, renal failure and veno-occlusive disease, as well as a 9% mortality. Siegert et al reported that at doses of carboplatin

 © 2001 Cancer Research Campaign

1.5 g/m$^2$, etoposide 2.4 g/m$^2$ and ifosfamide 10 g/m$^2$ (HD-CEI) WHO grade III/IV haematotoxicity occurred in 100%, nausea in 100% and diarrhoea in 30% and hepatotoxicity in 10% of patients. All patients developed granulocytopenic fever. 3% of patients died of treatment-related complications.

In our pilot study amifostine has also shown to significantly ameliorate the gastrointestinal side effects of HD-VIC chemotherapy such as stomatitis, delayed nausea and vomiting, and severe diarrhoea/enteritis. Acute nausea shortly after or during the application of amifostine was a manageable therapeutic problem with trifluorpromazine added prophylactically to a 5-HT$_3$-receptor antagonists plus dexamethasone. The reduced rate of delayed nausea/vomiting and less stomatitis in the amifostine arm even resulted in a lower rate of subsequently applied antiemetic medications and parenteral nutritions.

Amifostine has previously been demonstrated to reduce chemotherapy-induced neutropenia and cumulative bone marrow toxicity of treatment with carboplatin, cyclophosphamide and cisplatin (Glover et al, 1986; Treskes et al, 1993; Budd et al, 1999). In the current series with prophylactic application of G-CSF granulocyte engraftment occurred nearly at the same time in both treatment arms. Thrombocyte engraftment was observed earlier in the amifostine arm compared to controls. The number of reinfused PBSC was comparable between both arms excluding a possible source of bias in this analysis. Even this moderately faster haematopoietic recovery in the amifostine arm was associated with a fewer number of days with fever > 38.5˚C and a lower requirement of i.v. antibiotics.

The calculation of treatment costs has become important in the oncological setting, particularly when introducing drugs for supportive care (Bennett et al, 1994, 1999; Glasziou and Mitchell, 1996; Fayers and Hand, 1999). Aspects of quality of life and economics are nowadays established separate endpoints in cancer trials. Agents that may result in acute and/or long-term benefits should thus be evaluated in detail. We have provided a clinical economic estimate of direct treatment costs during the in-patient period in the HDCT-setting from a third party payer view. It was not possible to consider treatment costs for possible long-term toxicities and indirect non-medical costs in this analysis. The reduction of HDCT-related acute toxicities by amifostine resulted in lower costs for supportive care medication and an earlier discharge of patients from the hospital. Savings of supportive care costs in the amifostine arm were Euro 1243 per patient. Taking into account the drug costs of amifostine (Euro 1783 per patient), treatment with amifostine resulted in incremental costs of Euro 540 per patient. Similar to the use of G-CSF in adult patients with acute myelogenous leukaemia with in-patient costs savings of US $2300 (Bennett et al, 1999), previous pharmaeconomic reports have indicated that amifostine has a favourable clinical and cost–utility profile in the treatment of advanced ovarian cancer compared to other medical therapies such as autologous bone marrow transplantation for relapsed Hodgkin's disease or acute non-lymphocytic leukaemia or adjuvant tamoxifen in pre-menopausal oestrogen receptor-negative women (Detsky and Naglie, 1990).

In summary, the results from this randomized pilot study indicate that amifostine can ameliorate the acute side effects of HD-VIC chemotherapy with overall additional costs of approximately Euro 550 per patient. Thus, a larger prospective trial needs to address the definitive degree at which amifostine may increase the safety of the widely used HDCT regimens based on carboplatin, etoposide and ifosfamide and possibly other HDCT regimens. Whether the reduction of the level of acute toxicity is transferred into a lower degree of long-term toxicity and further costs savings has to be determined in consecutive trials.

### REFERENCES

Attal M, Harousseau JL, Stoppa AM, Sotto JJ, Fuzibet JG, Rossi JF, Casassus P, Maisonneuve H, Facon T, Ifrah N, Payen C and Bataille R (1996) A prospective, randomized trial of autologous bone marrow transplantation and chemotherapy in multiple myeloma. *N Engl J Med* **335**: 91–97

Barnett MJ, Coppin CM, Murray N, Nevill TJ, Reece-DE, Klingemann-HG, Shepherd-JD, Nantel-SH, Sutherland-HJ and Phillips-GL (1993) High dose chemotherapy and autologus bone marrow transplantation for patients with poor prognosis nonseminomatous germ cell tumours. *Br J Cancer* **68**: 594–598

Bennett CL, Armitage JL, Buchner D and Gulati S (1994) Economic analysis in phase III clinical cancer trials. *Cancer Invest* **12**: 336–342

Bennett CL, Stinson TJ, Tallman MS, Stadtmauer EA, Marsh-RW, Friedenberg W, Lazarus HM, Kaminer L, Golub RM and Rowe JM (1999) Economic analysis of a randomized placebo-controlled phase III study of granulocyte macrophage colony stimulating factor in adult patients (> 55 to 70 years of age) with acute myelogenous leukemia. *Ann Oncol* **10**: 177–182

Beyer J, Rick O, Weinknecht S, Kingreen D, Lenz K and Siegert W et al (1997) Nephrotoxicity after high dose carboplatin, etoposide and ifosfamide in germ-cell tumors: incidence and implications for hematologic recovery and clinical outcome. *Bone Marrow Transplant* **20**: 813–819

Broun ER, Nichols CR, Tricot G, Loehrer PJ, Williams SD and Einhorn LH (1991) High dose carboplatin/VP-16 plus ifosfamide with autologous bone marrow support in the treatment of refractory germ cell tumors. *Bone Marrow Transplant* **7**: 53–56

Budd GT, Ganapathi R, Wood L, Snyder J, McLain D and Bukowski RM (1999) Approaches to managing carboplatin induced thrombocytopenia: Focus on the role of amifostine. *Sem Oncol* **26**: 41–50, (suppl 7)

Calvert AH, Newell DR, Gumbrell LA, O'Reilly S, Burnell M, Boxall FE, Siddik ZH, Judson IR, Gore ME and Wiltshaw E (1989) Carboplatin dosage: prospective evaluation of a simple formula based on renal function. *J Clin Oncol* **7**: 1748–1756

Detsky AS and Naglie IG (1990) A clinician's guide to cost effectiveness analysis. *Ann Intern Med* **113**: 147–154

Eisenberg JM (1989) Clinical economics: A guide to the economic analysis of clinical practices. *JAMA* **262**: 2879–2886

Elias AD, Ayash LJ, Wheeler C, Schwartz G, Tepler I, Gonin R, McCauley M, Mazanet R, Schnipper L and Frei E (1995) Phase I study of high dose ifosfamide, carboplatin, etoposide with autologous hematopoietic stem cell support. *Bone Marrow Transplant* **15**: 373–379

Elias A, Richardsen P, Tretyakov O, Avigan A, Warren D, Arthur T, McCauley M, Wright J and Frei E (2000) Amifostine with high dose ifosfamide, carboplatin, and etoposide with hematopoietic stem cell support. *Proc Am Soc Clin Oncol* **19**: 51a (abstr. 197)

Fayers PM and Hand DJ (1999) Generalisation from phase III clinical trials: survival, quality of life, and health economics. *Lancet* **350**: 1025–1027

Fetscher S, Brugger W, Engelhardt R, Kanz L, Hasse J, Frommhold H, Lange W and Mertelsmann R (1999) Standard and high dose etoposide, ifosfamide, carboplatin, and epirubicin in 100 patients with small cell lung cancer: A mature follow up report. *Ann Oncol* **10**: 561–567

Fields KK, Elfenbein GJ, Lazarus HM, Cooper BW, Perkins JB, Creger RJ, Ballester OF, Hiemenz JH, Janssen WE and Zorsky PE (1995) Maximum tolerated doses of ifosfamide, carboplatin, and etoposide given over 6 days followed by autologous stem cell rescue: toxicity profile. *J Clin Oncol* **13**: 323–332

Gelmon K, Eisenhauer E, Bryce C, Tolcher A, Mayer L, Tomlinson E, Zee B, Blackstein M, Tomiak E, Yau J, Batist G, Fisher B and Iglesias J (1999) Randomized phase II study of high-dose paclitaxel with or without amifostine in patients with metastatic breast cancer. *J Clin Oncol* **17**: 3038–3047

Gianni AM, Bregni M, Siena S, Brambilla C, DiNicola M, Lombardi F, Gandola L, Tarella C, Pileri A, Ravagnani F, Valagussa P, Bonadonna G, Stern AC, Magni M and Caracciolo D (1997a) High dose chemotherapy and autologous bone marrow transplantation compared with MACOP B in aggressive B cell lymphoma. *N Engl J Med* **336**: 1290–1297

Gianni AM, Siena S, Bregni M, DiNicola M, Orefice S, Cusumano F, Salvadori B, Luini A, Greco M, Zucali R, Rilke F, Zambetti M, Valagussa P and Bonadonna G (1997b) Efficacy, toxicity, and applicability of high dose sequential chemotherapy as adjuvant treatment in operable breaset cancer with

© 2001 Cancer Research Campaign 

10 or more involved axillary nodes: five year results. *J Clin Oncol* **15**: 2312–2321

Glasziou PP and Mitchell AS (1996) Use of pharmacoeconomic data by regulatory authorities. In: *Quality of life and pharmacoeconomics in clinical trials*, Spiker B (ed) pp. 1141–1147. Lippinciott-Raven: Philadelphia, PA

Glover D, Glick JH, Weiler C, Hurowitz S and Kligerman MM (1986) WR 2721 protects against the hematologic toxicity of cyclophosphamide: a controlled phase II trial. *J Clin Oncol* **4**: 584–588

Goren MP, Wright RK, Horowitz ME and Pratt CB (1987) Ifosfamide-induced subclinical tubular nephrotoxicity despite mesna. *Cancer Treat Rep* **71**: 127–130

Haioun C, Lepage E, Gisselbrecht C, Bastion Y, Coiffier B, Brice P, Bosly A, Dupriez B, Nouvel C, Tilly H, Lederlin P, Biron P, Briere J, Gaulard P and Reyes F (1997) Benefit of autologous bone marrow transplantation over sequential chemotherapy in poor risk aggressive non Hodgkin's lymphoma: Updated results of the prospective study LNH87 2. *J Clin Oncol* **15**: 1131–1137

Hartmann JT, Kanz L and Bokemeyer C (1999a) Diagnosis and treatment of patients with testicular germ cell cancer. *Drugs* **58**: 257–281

Hartmann JT, Kollmannsberger C, Kanz L and Bokemeyer C (1999b) Platinum organ toxicity and its possible prevention in patients with testicular cancer. *Int J Cancer* **83**: 866–869

Hartmann JT, Knop S, Fels LM, van Vangerow A, Stolte H, Kanz L and Bokemeyer C (2000a) The use of reduced doses of amifostine to ameloriate nephrotoxicity of cisplatin/ifosfamide-based chemotherapy in patients with solid tumors. *Anti-Cancer Drugs* **11**: 1–6

Hartmann JT, Fels LM, Knop S, Stolte H, Kanz L and Bokemeyer C (2000b) A randomized trial comparing the nephrotoxicity of cisplatin/ifosfamide-based combination chemotherapy with or without amifostine in patients with solid tumors. *Invest New Drugs* **18**: 281–289

Hartmann JT, Fels LM, Franzke A, Knop S, Renn M, Maeß B, Panagiotou P, Lampe H, Kanz L, Stolte H and Bokemeyer C (2000c) Evaluation of the acute nephrotoxicity of cisplatin- and high dose carboplatin based combination chemotherapy. *Anticancer Research* **20**: 3767–3774

Kemp G, Rose P, Lurain J, Berman M, Manetta A, Roullet B, Homesley H, Belpomme D and Glick J (1996) Amifostine pretreatment for protection against cyclophosphamide induced and cisplatin induced toxicities: results of a randomized control trial in patients with advanced ovarian cancer. *J Clin Oncol* **14**: 2101–2112

Linch DC, Winfield D, Goldstone AH, Moir D, Hancock B, McMillan A, Chopra R, Milligan D and Hudson GV (1993) Dose intensification with autologous bone marrow transplantation in relapsed and resistant Hodgkin's disease: results of a BNLI randomized trial. *Lancet* **341**: 1051–1054

Margolin K, Doroshow JH, Ahn C, Hamasaki V, Leong L, Morgan R, Raschko J, Shibata S, Somlo G and Tetef M (1996): Treatment of germ cell cancer with two cycles of high dose ifosfamide, carboplatin, and etoposide with autologous stem cell support. *J Clin Oncol* **14**: 2631–2637

Philip T, Guglielmi C, Hagenbeek A, Somers R, van der Lelie H, Bron D, Sonneveld P, Gisselbrecht C, Cahn JY, Harousseau JL, Coiffier B, Biron P, Mandelli F and Chauvin F (1995) Autologous bone marrow transplantation as compared with salvage chemotherapy in relapses of chemotherapy sensitive non Hodgkin's lymphoma. *N Engl J Med* **333**: 1540–1545

Rizzieri DA, Vredenburgh JJ, Jones R, Ross M, Shpall EJ, Hussein A, Broadwater G, Berry D, Petros WP, Gilbert C, Affronti ML, Coniglio D, Rubin P, Elkordy M, Long GD, Chao NJ and Peters WP (1999) Prognostic and predictive factors for patients with metastatic breast cancer undergoing aggressive induction therapy followed by high-dose chemotherapy with autologous stem-cell support. *J Clin Oncol* **17**: 3064–3074

Rosti G, Albertazzi L, Salvioni R, Pizzocaro G, Cetto GL, Bassetto MA and Marangolo M (1992) High dose chemotherapy supported with autologous bone marrow transplantation (ABMT) in germ cell tumors: a phase two study. *Ann Oncol* **3**: 809–812

Russell LB, Gold MR, Siegel JE, Daniels N and Weinstein MC (1996) The role of cost effectiveness analysis in health and medicine. *JAMA* **276**: 1172–1177

Savarese DEM, Hsieh C and Stewart FM (1997) Clinical impact of chemotherapy dose escalation in patients with hematologic malignancies and solid tumors. *J Clin Oncol* **15**: 2981–2995

Siegert W, Beyer J, Strohscheer I, Baurmann H, Oettle H, Zingsem J, Zimmermann R, Bokemeyer C, Schmoll HJ and Huhn D (1994) High dose treatment with carboplatin, etoposide, and ifosfamide followed by autologous stem-cell transplantation in relapsed or refractory germ cell cancer: a phase I/II study. The German Testicular Cancer Cooperative Study Arm. *J Clin Oncol* **12**: 1223–1231

Treskes M and van der Vijgh W (1993) WR2721 as a modulator of cisplatin- and carboplatin-induced side effects in comparison with other chemoprotective agents: a molecular approach. *Cancer Chemother Pharmacol* **33**: 93–106

van Dam FSAM, Schagen SB, Muller MJ, Boogerd W, von der Wall E, Fortuyn MED and Rodenhuis S (1998) Impairment of cognitive function in women receiving adjuvant treatment for high risk breast cancer: High dose versus standard dose chemotherapy. *J Natl Cancer Inst* **90**: 210–218

Wadler S, Haynes H, Beitler JJ, Goldberg G, Holland JF, Hochster H, Bruckner H, Mandeli J, Smith H and Runowicz C (1993) Management of hypocalcemic effects of WR2721 administered on a daily times five schedule with cisplatin and radiation therapy. The New York Gynecologic Oncology Group. *J Clin Oncol* **11**: 1517–1522

Wagstaff AJ, Ward A, Benfield P and Heel RC (1989) Carboplatin. A preliminary review of its pharmacodynamic and pharmacokinetic properties and therapeutic efficacy in the treatment of cancer. *Drugs* **37**: 162–190

Wilson WH, Jain V, Bryant G, Cowan KH, Carter C, Cottler Fox M, Goldspiel B Steinberg SM, Longo DL and Wittes RE (1992) Phase I and II study of high dose ifosfamide, carboplatin, and etoposide with autologous bone marrow rescue in lymphomas and solid tumors. *J Clin Oncol* **10**: 1712–1722

Wright JE, Elias A, Tretyakov O, Holden S, Andersen J, Wheeler C, Schwartz G, Antman K, Rosowsky A and Frei E (1995) High dose ifosfamide, carboplatin, and etoposide pharmacokinetics: correlation of plasma drug levels with renal toxicity. *Cancer Chemother Pharmacol* **36**(4): 345–351

Zittoun RA, Mandelli F, Willemze R, de Witte T, Labar B, Resegotti L, Leoni F, Damasio E, Visani G and Papa G (1995) Autologous or allogeneic bone marrow tranplantation compared with intensive chemotheraphy in acute myelogenous leukemia. Europeon Organization for Research and Treatment of Cancer (EORTC) and the Gruppo Italiano Malattie Ematologiche Maligne dell'Adulto (GIMEMA) Leukemia co-opearative Arms. N Engl. J Med 332: 217–223

 *© 2001 Cancer Research Campaign*
